# Interactions between Global Health Initiatives and Country Health Systems: The Case of a Neglected Tropical Diseases Control Program in Mali

**DOI:** 10.1371/journal.pntd.0000798

**Published:** 2010-08-17

**Authors:** Anna Cavalli, Sory I. Bamba, Mamadou N. Traore, Marleen Boelaert, Youssouf Coulibaly, Katja Polman, Marjan Pirard, Monique Van Dormael

**Affiliations:** 1 Public Health Department, Institute of Tropical Medicine, Antwerp, Belgium; 2 Direction Nationale de la Santé, Ministère de la Santé, Bamako, Mali; 3 Parasitology Department, Institute of Tropical Medicine, Antwerp, Belgium; London School of Hygiene & Tropical Medicine, United Kingdom

## Abstract

**Background:**

Recently, a number of Global Health Initiatives (GHI) have been created to address single disease issues in low-income countries, such as poliomyelitis, trachoma, neonatal tetanus, etc.. Empirical evidence on the effects of such GHIs on local health systems remains scarce. This paper explores positive and negative effects of the Integrated Neglected Tropical Disease (NTD) Control Initiative, consisting in mass preventive chemotherapy for five targeted NTDs, on Mali's health system where it was first implemented in 2007.

**Methods and Findings:**

Campaign processes and interactions with the health system were assessed through participant observation in two rural districts (8 health centres each). Information was complemented by interviews with key informants, website search and literature review. Preliminary results were validated during feedback sessions with Malian authorities from national, regional and district levels. We present positive and negative effects of the NTD campaign on the health system using the WHO framework of analysis based on six interrelated elements: health service delivery, health workforce, health information system, drug procurement system, financing and governance. At point of delivery, campaign-related workload severely interfered with routine care delivery which was cut down or totally interrupted during the campaign, as nurses were absent from their health centre for campaign-related activities. Only 2 of the 16 health centres, characterized by a qualified, stable and motivated workforce, were able to keep routine services running and to use the campaign as an opportunity for quality improvement. Increased workload was compensated by allowances, which significantly improved staff income, but also contributed to divert attention away from core routine activities. While the campaign increased the availability of NTD drugs at country level, parallel systems for drug supply and evaluation requested extra efforts burdening local health systems. The campaign budget barely financed institutional strengthening. Finally, though the initiative rested at least partially on national structures, pressures to absorb donated drugs and reach short-term coverage results contributed to distract energies away from other priorities, including overall health systems strengthening.

**Conclusions:**

Our study indicates that positive synergies between disease specific interventions and nontargeted health services are more likely to occur in robust health services and systems. Disease-specific interventions implemented as parallel activities in fragile health services may further weaken their responsiveness to community needs, especially when several GHIs operate simultaneously. Health system strengthening will not result from the sum of selective global interventions but requires a comprehensive approach.

## Author Summary

Prevention of neglected tropical diseases was recently significantly scaled up in sub-Saharan Africa, protecting entire populations with mass distribution of drugs: five different diseases are now addressed simultaneously with a package of four drugs. Some argue however that, similarly to other major control programs dealing with specific diseases, this NTD campaign fails to strengthen health systems and might even negatively affect regular care provision. In 2007, we conducted an exploratory field study in Mali, observing how the program was implemented in two rural areas and how it affected the health system. At the local level, we found that the campaign effects of care delivery differed across health services. In robust and well staffed health centres, the personnel successfully facilitated mass drug distribution while running routine consultations, and overall service functioning benefitted from programme resources. In more fragile health centres however, additional program workload severely disturbed access to regular care, and we observed operational problems affecting the quality of mass drug distribution. Strong health services appeared to be profitable to the NTD control program as well as to general care.

## Introduction

Since 2000, global health initiatives (GHIs) have become a dominant international aid strategy, drawing on effective methods to control specific diseases, and accounting for a substantial increase of resources for global health [1]. Very soon, however, concerns emerged that, beyond anticipated benefits for targeted diseases, GHIs might erode health systems' capacity to respond to general health needs [2]–[7]. Early criticisms of GHI included the distortion of national policies as well as the creation of parallel bodies and processes burdening the health system [8]. Conversely, GHIs realized rapidly that their intervention capacity was limited by countries' weak health systems [6].

While it is now acknowledged that GHIs and health systems are influencing each other [8], [9] ^,^ health systems and GHIs advocates still tend to have divergent views, partly framed in the long-standing horizontal-vertical discussion [10]. A WHO collaborative group was assigned in 2008 to assess the interactions between health systems and GHIs [9], and its findings were discussed during a policy dialogue meeting in Venice in June 2009 [11]. The report draws attention to the paucity of evidence to help understanding the interactions between GHIs and health systems. So far, most studies have dealt with global interventions in the field of HIV/AIDS control [8]. These research results may however not be applicable to other GHIs, as distinct objectives, policies, structures and operational processes of GHIs are likely to produce distinct effects on health systems [9]. Another limitation is that most studies focus on national level, while empirical evidence at point-of-delivery level remains particularly narrow [8], [9].

In recent years, growing awareness of NTDs, coupled with the availability of relatively low-cost control strategies, have led to important new global initiatives, including the WHO Neglected Tropical Disease Program, the Schistosomiasis Control Initiative (SCI), the Global Network for Neglected Tropical Diseases (GNNTD), the Neglected Tropical Disease Initiative (NTDI), and others [12]–[14]. The emphasis of the current global NTD control strategy is on mass drug administration. Based on geographic overlap and co-endemicity of NTDs, it addresses simultaneously up to five NTDs (lymphatic filariasis, onchocerciasis, schistosomiasis, soil-transmitted helminthiasis, and trachoma) with a package of 4 drugs (ivermectin or diethylcarbamazine, praziquantel, albendazole or mebendazole, and azithromycin). Mass chemotherapy conducted over successive years is expected to eliminate or reduce NTDs to a prevalence rate at which they no longer pose a threat to public health. The case for efficiency of the intervention is based on the “integration” of 5 diseases, but also on the fact that drugs are donated by pharmaceutical companies or available as generics, and distributed by community volunteers [15].

Mali, which already had experience with distinct campaigns for trachoma and schistosomiasis, was the first country to implement this integrated NTD control program in 2007 with USAID financial support. Since the knowledge on possible side effects of drug combinations was deemed insufficient, drugs were distributed serially for precautionary reasons, in a total period of 7 weeks between April and June 2007. Each single drug distribution was followed by a 2 weeks break (week 1: azitromicine, week 4: albendazole and ivermectine, week 7: praziquantel). For temporary financial constraints, the campaign was launched only in 3 regions, the plan for the rest of the country being postponed to end 2007. The campaign occurred simultaneously in the 3 regions and concerned 24 districts; in some of these districts, this coincided with a Vitamin A campaign.

The present study analyzes interactions between the NTD Control Program and Mali's health system, with a special focus on the district and service delivery level. Health districts in Mali are based on a network of health centers, each covering a defined health area; a typical health centre is staffed by a qualified nurse and 2 to 4 auxiliaries. A district executive team runs the referral hospital and provides technical support to the health centers.The aim of this exploratory study was to assess the program implementation processes on the field, and to identify plausible positive and negative effects for the health care system.

## Materials and Methods

### Data collection and analysis

To gain more insight into the interactions between the NTD campaign and the local health system in Mali, we carried out an exploratory qualitative study, which is a common approach to study situations with little prior knowledge and few definite hypotheses [16].

We chose to observe NTD campaign interactions with health services in reasonably well functioning settings, in order to avoid that our findings could be attributed to major failures of the local health care system. Using ‘purposeful’ sampling [16], [17], we identified two rural districts from two different Regions that were typical of “good” rural districts in terms of output indicators (utilization rate for curative services and coverage rates for preventive activities).

Our approach combined three standard qualitative data collection methods, i.e. participant observation, in depth interviews with key informants and document analysis [16]. Interviews are valuable to gain information on feelings, thoughts and opinions, but less useful to describe events, behaviour or settings, as responses tend to be distorted by personal biases, lack of awareness, recall errors or selective accounts [16]. Participant observation is more appropriate to understand context issues, events and processes, but also has limitations including atypical behaviour of those being observed and selective perceptions of the observer [16]. Official documents provide a range of helpful information, including on planned processes and their rationale, but are selective and do not necessarily reflect actual processes. By using different methods we intended to compensate for limitations of each of them and to cross- check and triangulate our findings [16], [17].

Participant observation was conducted during two weeks in May – June 2007 by a researcher (AC) with a public health background, who was familiar with health systems in various sub-Saharan countries. In each of the two districts, she accompanied the district medical officer and/or health centre staff in their follow up of campaign activities at health centre and community level. This allowed for observations of mass drug administration in 16 health areas (8 areas per district); the selection of these areas was opportunistically determined by the district's agenda. Observation focused primarily on contextual issues, on procedures (e.g. task allocation, place of distribution, contents of information, dosage) and on behaviour of staff, drug distributors and community members. During observations, the researcher also used situational conversations, i.e. asking on-the-spot questions and discussing with district authorities and health centre staff in a naturalistic and informal way [18], [19]. Besides routine drug administration, participant observation including situational conversations also entailed a “training of trainers” session for district health teams at Regional level, two district executive team meetings, and a community meeting.

Structured interviews were not conducted at local level due to heavy time constraints for staff during the campaign. But in depth interviews were conducted with key informants, including ten Ministry of Health officers and ten representatives of support agencies acquainted with the Malian health system. Key informants were identified through snowball sampling [16]–[17]. These interviews sought to elicit information on campaign processes which could not be directly observed - such as decision making at national level, planning and financing - and to explore informants' views on interactions between the NTD control program and local health services.

Further information was collected by consulting Malian official documents, website search and literature review. We sought to triangulate the data as much as possible and to check the same information at different sources.

Observation and interview data were recorded in field note transcripts that were reviewed independently by two researchers. The study being exploratory, categories for analysis were largely inductive, though some were based on interactions of other GHIs with health systems reported in the literature [2]–[7]. They included implementation processes (training, drug procurement and distribution, monitoring), task distribution, positive and negative effects on service delivery, and decision making processes. Throughout these issues we looked for emerging recurrent patterns and variations among situations and/or informants.

Results were validated and complementary information was generated during three feedback sessions held in November 2007 with health authorities at peripheral, regional and national level.

### Ethics statement

Most data are based on field observation which had no potential harmful effects on patients or other vulnerable persons. Data collection was complemented by in depth interviews with twenty key informants. We asked about their opinions which were mostly public. Nevertheless we asked for oral consent after explaining the purpose and methods of our study, and ensured confidentiality, as some of the key informants who were high officials of the MOH or international organizations preferred not to be identified. This consent was witnessed by at least one person other than the principal investigator. No written consent of key informants was asked, as this would have been unusual in the context and might have biased the interview process, otherwise quite informal. Another ethical concern for this health systems research was governance: the national authorities of the Ministry of Health in Mali gave permission, regional and district authorities were supportive of this research, and provisional results were shared and discussed with authorities before broader dissemination. Potential ethical issues were examined with the head of the IRB of ITM (Antwerp) and it was decided that there was no need for a full review. Neither was it required under the Malian legislation (see law n°2009/63/4L), which rules biomedical research but not health systems research, and was not applicable at the time of the study.

## Results

Our results are presented according to the conceptual framework as presented by the WHO Maximizing Positive Synergies Collaborative Group [9]. We examine both positive and negative effects of the NTD control program on health service delivery, health workforce, health information system, supply management system, financing, and governance. These effects are summarized in Table 1.

**Table 1:** Effects of interactions between NTD control and health system.

Point of interaction	Positive effects	Negative effects
**Health service delivery**	▪ Increased access to preventive chemotherapy▪ In robust health centres: campaign as opportunity to strengthen health centre capacity and responsiveness	▪ Missed opportunities for curative care▪ In fragile health centres: absence of qualified staff from the centre = general activities interruption; operational problems in campaign implementation▪ The prospect of free drug distribution may affect routine health service utilisation
**Health workforce**	▪ Refresher course on NTDs▪ Allowances for district medical officer (increase of 81% monthly salary), health centre nurse (increase of 46% monthly salary) and community volunteers▪ Allowances possibly affecting staff retention	▪ Increased workload for district staff and health centre staff▪ Training benefits may be low when consisting in transmission of known information▪ Allowances possibly distracting of staff's attention from core activities▪ Doubts about sustainability of allowances to community volunteers
**Health information system**	▪ Availability of NTD treatment coverage data▪ Census data useful for purposes other than NTD control	▪ Parallel monitoring and evaluation system (total 25 new forms at district level); specific timetable for reporting
**Supply management system**	▪ Increase of NTD drugs' availability at country level	▪ Parallel drug supply system: hiring of special transport (trucks)▪ Imbalance: some drugs available for mass distribution are not available for routine curative care
**Financing and Governance**	▪ Increased availability of funds▪ Strengthening of ongoing country efforts in NTD control▪ Design of a single NTD control plan; stimulation of coordination between programs	▪ Limited margins of manoeuvre: earmarked budgets and funding▪ Limited and selective institutional strengthening▪ Doubts on long term sustainability▪ Accumulation of donors conditions distorting national strategic orientations▪ Top-down decision-making interfering with district regional and district plans and calendars

### Health service delivery

Access to mass chemotherapy for targeted NTDs clearly improved according to all interviewees. Some informants however regretted that the control program only included drug distribution and some health education on NTDs, and did not address other NTD disease control strategies, such as curative care (e.g. eye surgery for trachoma) or sanitation.

Several informants also criticized the high priority given to targeted diseases, while more common health problems received little attention; they worried about the campaign mobilising energy and diverting staff's attention from routine care delivery. These interview results were in line with our direct field observations: routine care was cut down or even totally interrupted in most health centres observed, as a consequence of health centre nurses being absent from their station and not being replaced by other staff. During the first round of the NTD control program, head nurses were required to devote 10 full working days for program-related training and supervision, in addition to monitoring and drug supply activities (Table 2). The mass distribution schedule also required health centre staff to postpone or reorganise planned routine outreach immunisation sessions.

**Table 2:** Absences of head nurses due to NTD campaign-related activities.

Activity	N° of working days
Participation training of trainers	4
Training community distributors	3
Supervision of the distribution	3
Drugs need definition and management	Not measurable
Drugs adverse effects monitoring	Not measurable
Data collection and transmission	Not measurable
Community mobilization	Not measurable
Restitution phase	Not measurable
TOTAL	>10

Some informants thought that, in a context of low service utilisation, the campaign was at least a way to bring services closer to underserved populations. Observation however did not suggest that the campaign had positive effects on nontargeted services. We observed missed opportunities for curative care: children queuing for NTD prophylactic drugs and presenting obvious other illnesses and need for care (e.g. abscesses or trauma) were not identified as such by the attending staff.

Not all health centers responded similarly to these interferences: 2 of the 16 health centers managed to keep their curative consultations and immunization services running normally and to use the campaign to support the overall development of their health centre. For instance, one nurse passed his NTD training on to other team members, another took advantage of NTD training of community volunteers to discuss other health issues than the targeted NTDs, and supervision of the campaign at village level became an opportunity for health education on other topics. These 2 health centres differed from the others in terms of human resources: both were well staffed and had no vacant positions. They were managed by a qualified nurse, who was on the job for over 5 years, and reputed for dynamism, professionalism and leadership, both at regional and central level. Moreover, utilisation rates in these centres were above the national average (>0.20 new cases/inhabitant/year), preventive coverage rates were considered good (>75%), and they benefited from a supportive community organisation.

Disparities between health centres were also seen with respect to the health centre capacity to implement the NTD control program: operational problems were observed in all health centres, except for the two more robust ones. These problems included errors in the population census, poor community mobilisation, drug dosing errors and omission of side effect monitoring. While these problems were not captured by the monitoring system of the NTD campaign, which indicators were limited to treatment and geographical coverage, they nevertheless suggest quality problems in campaign implementation processes.

Finally, several interviewees highlighted possible negative effects of free distribution of drugs on care seeking: as sick patients have to pay for drugs in routine circumstances: patients might, so they feared, wait for a next edition of the campaign rather than seek care.

### Health workforce

While mass drug distribution itself relied on community volunteers, the NTD campaign nevertheless implied increased workload for both district and health centre staff to ensure drug supply, campaign monitoring, training and supervision.

Informants thought that the training component was a positive effect of the program. A training cascade was organized, starting with a “training of trainers” where national program coordinators trained district authorities. The cascade continued with district authorities training health centre nurses, who trained community volunteers. The training consisted in transmission of information on epidemiology, diagnosis and treatment of each of the targeted diseases; several participants said it was a repetition of previous training sessions.

Training and supervision activities entitled staff to receive allowances, which represented approximately an 80% increase of a district medical officer monthly salary (increase of 122 000 F CFA for an average salary of 150 000 F CFA), and a 45% increase of a health centre nurse salary (increase of 46 000 F CFA for an average salary of 100 000 F CFA). Some informants considered these incentives as contributing to the motivation and retention of health staff, but others reported that these allowances distracted staff's attention from their core activities, for which no extra allowances were provided.

Allowances were also given to community volunteers. Most informants considered this as necessary to attract volunteers. We witnessed a local dispute around the selection of volunteers, suggesting that becoming a volunteer was much sought after. Still, informants worried about the sustainability of allowances for volunteers (which were supposed to be paid by the communities themselves after the first round of the NTD control program), as well as about the inconsistency of allowance amounts between various donors, generating growing demands from the side of volunteers.

### Health information system

Preparatory to mass drug distribution, census data were collected which can be used for purposes other than NTD control. Improved information on NTD treatment coverage was also made available.

The NTD campaign however introduced a parallel monitoring and evaluation system. At district level, 12 new forms were introduced for drug supply management, 15 new forms for monitoring and supervision of campaign activities, and 1 new form for clinical complications of filariasis (Table 3). For each drug distributed, one report per village, one per health centre and one per district was required. Following donor instructions, a specific timetable for reporting was installed: village data were processed daily at health centre level, and transmitted to district level. Districts reported campaign results weekly to the regional level.

**Table 3:** New forms for NTD campaign-related activities.

Type of form	Description	Number introduced
Drug supply management	Inventory cards	3
	Stock changes	3
	Drug reception	3
	Drugs distributed	3
Drug distribution monitoring	District reports	3
	Area reports	3
	Village reports	3
Supervision	Community	3
	District	3
Report of clinical complications for Lymphatic Filariasis		1
**TOTAL**		**25**

### Supply management system

NTD campaign drugs were mostly donated by pharmaceutical companies, which increased their availability for preventive chemotherapy nationwide. But a parallel drug procurement system was established to ensure rapid distribution of drugs from national to regional and district level. As storage space at national and regional level was insufficient, trucks were especially rented for the occasion. Drug management forms and processes, distinct from the national ones, were created specifically for campaign implementation (see Table 3). Besides, some informants stressed imbalances in drug availability: while Azitromicine was distributed at no cost during the campaign, patients suffering from trachoma had no access to this drug in routine conditions, as it was substituted by payable tetracycline ointment. One local informant reported complaints from the community on this issue.

### Financing

According to official documents [20], the provisional budget for mass drug administration – cost of drugs not included - was approximately US $ 12 million spread over 5 years, most of which was financed by USAID, with complements from other agencies. This budget covered drug distribution (29% of the budget), training of staff and volunteers (26%), supervision and evaluation (17%), drug procurement (9%), health education (7%), institutional strengthening (10%) and intersectoral collaboration (2%). Not included in the budget were State supported staff salaries and infrastructure used for the campaign. Institutional strengthening consisted mostly in intervention related equipment and staff, and several informants considered that the budget left no room for institutional strengthening beyond campaign related needs. Investments in general equipment were limited to motorbikes for districts; the increase of storage capacity at national level was not approved, nor was the acquisition (rather than rental) of trucks for drug procurement, which several informants regretted. Though acknowledging the relevance of the intervention, they expressed concerns about longer-term financing for sustainable campaign results.

### Governance

Though the decision to implement the program was taken by Malian authorities, several informants considered that national authorities had little space for negotiation, as most decisions were taken at supranational level by donors and their grantees. Indeed USAID financing was not directly allocated to the country, but to sub-grantees which, in the case of Mali were the International Trachoma Initiative (ITI), replaced by Helen Keller International at the end of 2007.

A steering group with coordination and decision-making power was set up in parallel to the existing coordination structures in the Malian Ministry of Health. It included MoH officials and ITI staff. The strategic NTD control plan for 2007–2011 was designed at National Health Directorate level. Several informants considered the design of a single NTD control plan as a positive effect of the program, as it stimulated coordination between previously standalone program coordinators. However, the strategic plan had to adapt to donor and grantees requirements. Predefined strategies, earmarked funding and budgets kept tight for the sake of demonstrating efficiency left only limited margins of maneuver.

Several informants were also critical about distorting effects of the program on national priorities. They recognized the importance of NTDs and Mali's longstanding experience with mass treatment targeting distinct NTDs: onchocerchiasis since 1988, trachoma since 2004, schistosomiasis since 2005, and attempts to integrate mass treatment for onchocerchiasis, filariasis and helminthiasis started in 2005. Donor websites emphasize that the program met ongoing country efforts and contributed to scale them up at national level. But informants also reported that these ongoing efforts partly resulted from previous external financing opportunities as well. They considered that the accumulation of donor conditions was hampering resource allocation following national strategic orientations.

Finally, the top-down implementation process of the NTD campaign was felt by most informants to contradict local district leadership, central to Malian health policy (PRODESS II), and to interfere with planned activities. Indeed, as information concerning the campaign reached regional and district authorities with short notice, district authorities had to modify or adjourn their calendar of health centre supervisions with no space for negotiation.

## Discussion

This study is the first to address interactions between the integrated NTD control program and a country health system. It provides insights on positive and negative effects of the integrated NTD control program at point-of-delivery and on district systems. A previous study documented community resistance to free drug distribution for schistosomiasis and soil transmitted helminths in Uganda, but did not address health system effects [21].

A few limitations of this study should not remain unnoticed. Data collection was inevitably influenced by the presence of the researcher and relations between her and people in the field [17]. It is however plausible that investigator effects led authorities and staff to show the best of their performance and refrain from critical comments: biases are more likely to minimise rather than maximise problems. Furthermore this qualitative study was based on a limited number of interviews and contextualised observation units, and not intended to be generalisable as in quantitative research, though readers may assess their applicability to their own settings [17] Purposeful sampling [16]–[17] does not provide guarantees that the districts and health centres observed are typical of the country. As we selected “better performing” districts in terms of output indicators, we assume that campaign related problems are not more severe in these districts than elsewhere in the country, but this needs to be probed. Another reason for caution in transferring our findings to other settings or countries is that the Malian campaign was the very first NTD control program integrating five diseases and may have suffered from startup problems, avoided in further editions. As most USAID-supported NTD campaigns are based on similar principles and processes as in Mali, our study however provides plausible hypotheses to be tested in other contexts. The purpose of our study was exploratory, which implies that more research is needed, both qualitative and quantitative. We suggest that our findings are helpful in framing further research questions.

A large part of current understanding of interactions between GHI and health systems is based on HIV/AIDS related programs. Though the NTD control program appears as a “small” GHI compared to others such as Global Fund or PEPFAR [1], its analysis brings new insights to the ongoing debate.

The NTD control program, like other preventive programs, focuses on protection rather responsiveness to patient demand [22]. This distinguishes it from HIV/AIDS related GHIs scaling up access to ARV treatment for individual patients. We found that the integrated NTD program did not include curative care for NTDs, but also that extra workload and staff absences for program purposes disrupted access to general curative care at health centre level. A Bill and Melinda Gates Foundation study [23] reported extra workload for district staff in Angola and Tanzania resulting from donor requirements, but did not assess effects on service delivery. Evidence on effects of workload generated by GHI on access to health centre care is so far extremely limited [24].

A key finding of our study is the emerging differentiation between health centres within the same district in their capacity to cope with program's interferences. Only the most resilient services, characterized by a qualified, stable and motivated workforce, managed to maintain routine activities, and even to use the program as an opportunity for overall quality improvement. This finding is consistent with the notion that positive effects of GHIs are more likely to occur when the health system is robust [9], [25]. But it expands it from country health system to district and health service level, emphasizing the importance of human resources for robustness of services. Future research should further explore the relation between human resources characteristics and absorption capacity of programs at health service level. Indeed, this might bear consequences for the adaptation of programs to specific local health systems and services.

Other findings of our research are coherent with known effects of other GHIs on health systems and show a mixture of positive and negative effects. Like other programs, uptake in targeted services was scaled up, but duplications occurred, especially for drug procurement and monitoring and evaluation [8]–[9]; these parallel systems, meant to improve campaign efficiency, increased workload and total costs for the health system. Also like other GHIs, the NTD control program influenced priority setting [7]: pressure to absorb donated drugs and reach short term chemotherapy coverage results contributed to the distraction of energies away from other NTD control strategies such as treatment and sanitation, and more generally from overall health systems strengthening.

From a health systems perspective, the question is however not so much whether the balance of a specific GHI is positive or negative. The problem for Mali, as for other countries, is the cumulative effect of a large number of GHIs and other campaigns, each with implications at all levels. Besides NTD campaigns, Malian health services also run National Immunisation Days, Vitamin A and bed-net distribution as well as eradication or elimination campaigns of polio, tetanus or yellow fever. An estimation of time spent outside the health centre by head nurses of a Malian rural district in 2006 showed absences reaching 54% of working days; half of this time was dedicated to campaigns and trainings linked to vertical programs [24]. As the head nurse is usually the only staff qualified to provide first line curative care in Mali, disruptions in consultation schedules erode service responsiveness and community's confidence in their local health centre [26]. Another cumulative effect is the growing mobilisation of communities to meet top down defined targets, to the expense of an empowerment approach to community participation [21], [27].

The need for health system strengthening is increasingly acknowledged, also by promoters of integrated NTD control [28]. Most GHIs claim to contribute to health system strengthening with additional resources and capacity strengthening, but these interventions are mostly selective, targeting those system functions essential for implementation of their own program [22]. This was also the case for the Malian NTD control program. The prospect of adding vitamin A, bed nets and vaccines to the present campaign model [28] might contribute to the improvement of the protective function of health systems, but not to their responsiveness to population's demand for curative care, which may be even further undermined [22].

The control of NTDs in vulnerable communities is a necessity. But so is health systems strengthening, in order to respond adequately to other health problems and to ensure sustainable achievements, including of NTD control. A major challenge is how to engage in disease control – NTD and other diseases - without negatively impacting on existing health systems. Increased knowledge on interactions with the health system is needed to allow GHIs to plan for positive effects and alleviate potential negative effects. Presently, short term and quick win interventions are given priority, but more long term strategies are also needed. Health system strengthening should rely on country-specific development plans aligned with national policy, and requires a comprehensive approach across diseases and health problems and coordination among GHIs. For example, program specific in-service training should be organised in ways mitigating potential interruptions of service provision, but investments are also needed in pre-service education for qualified staff. The accumulation of program specific extra allowances, making targeted interventions more popular than routine activities [29], could gradually be replaced by comprehensive human resource management at national and district level. Parallel drug supply should be limited to exceptional emergencies, and investments redirected to reinforce national drug supply systems.

There are signs of GHIs learning from experience and gradually modifying some of their processes [8]. They also show increasing willingness to reduce fragmentation and to review processes [11]. Still the chaotic architecture for development assistance for health remains a major obstacle for health system strengthening. Progress towards effective and inclusive health systems will not result from the sum of selective GHI interventions.

## Supporting Information

Supporting Text S1 Translation of the manuscript into French by author Van Dormael.
(0.20 MB DOC)
