# Supplementary material for: Interactions between Global Health Initiatives and Country Health Systems: The Case of a Neglected Tropical Diseases Control Program in Mali
Source: PLoS Negl Trop Dis. 2010 Aug 17;4(8):e798. doi: 10.1371/journal.pntd.0000798 (PMC2923152; doi:10.1371/journal.pntd.0000798)
Supplement: Supporting Text S1 — Translation of the manuscript into French by author Van Dormael. (0.20 MB DOC) [file pntd.0000798.s001.doc]

Les interactions entre Initiatives Mondiales en matière de Santé et les systèmes nationaux de santé : le cas du programme de contrôle des Maladies Tropicales Négligées au Mali

Anna Cavalli1, Sory I Bamba 2, Mamadou N Traore 2, Marleen Boelaert1,

Youssouf Coulibaly2, Katja Polman3, Marjan Pirard1, Monique Van Dormael1*

1 Département de Santé Publique, Institut de Médecine Tropicale, Anvers, Belgique

2 Direction Nationale de la Santé, Ministère de la Santé du Mali

3 Département de Parasitologie, Institut de Médecine Tropicale, Anvers, Belgique

E-mail : mvdormael@itg.be

Citation : Cavalli A, Bamba SI, Traore MN, Boelaert M, Coulibaly Y, et al. (2010) Les interactions entre Initiatives Mondiales en matière de Santé et les systèmes nationaux de santé : le cas du programme de contrôle des Maladies Tropicales Négligées au Mali

Citation article en anglais : Cavalli A, Bamba SI, Traore MN, Boelaert M, Coulibaly Y, et al. (2010) Interactions between Global Health Initiatives and country health systems: the case of a Neglected Tropical Diseases control program in Mali

Abstract

**Contexte**

De nombreuses Initiatives Mondiales en matière de Santé (IMS) se sont développées ces dernières années dans des pays à faible revenu pour contrôler des maladies spécifiques telles que la poliomyélite, le trachome, le tétanos néonatal etc. On dispose de peu d’études empiriques sur les effets de telles IMS sur les systèmes de santé locaux. Cet article explore les effets positifs et négatifs de l’Initiative Mondiale pour le contrôle des Maladies Tropicales Négligées (MTN) - qui à travers la chimiothérapie préventive de masse combat cinq maladies tropicales négligées - sur le système de santé du Mali, où cette stratégie a démarré en 2007.

**Méthodes et résultats**

Nous avons documenté les processus de la campagne de masse et ses interactions avec le système de santé par observation participante dans deux districts ruraux (8 centres de santé chacun). Ces informations ont été complétées par des interviews avec des informateurs clés, des recherches sur sites web et une revue de littérature. Nous avons validé nos résultats préliminaires lors de sessions de feedback avec les autorités Maliennes aux niveaux nationaux, régionaux et de district. Nous présentons les effets positifs et négatifs de la campagne MTN sur le système de santé en utilisant le cadre d’analyse de l’OMS basé sur six éléments interdépendants: la dispensation des soins, le personnel de santé, le système d’information sanitaire, le système d’approvisionnement en médicaments, le financement et la gouvernance. Dans les services de santé, la charge de travail liée à la campagne a fortement perturbé et parfois totalement interrompu la dispensation des soins courants, les infirmiers s’absentant du centre pour des activités liées à la campagne. Seuls 2 des 16 centres de santé, caractérisés par un personnel qualifié, stable et motivé, ont poursuivi leurs activités principales et utilisé la campagne comme opportunité d’amélioration de la qualité. La charge de travail supplémentaire était compensée par des allocations qui amélioraient sensiblement les revenus du personnel, mais qui contribuaient aussi à détourner l’attention des activités de base habituelles. La campagne a amélioré la disponibilité des médicaments pour MTN au niveau du pays, mais les systèmes parallèles d’approvisionnement en médicaments et d’évaluation mis en place ont alourdi la charge de travail pour le système de santé local. Le budget de la campagne a peu financé le renforcement institutionnel du système de soins. Finalement, et bien que la campagne reposait au moins partiellement sur les structures nationales, les pressions pour absorber les médicaments offerts et atteindre rapidement des résultats en termes de couverture ont contribué à détourner les énergies d’autres priorités, y compris le renforcement général du système de santé.

**Conclusions**

Notre étude indique que des synergies positives entre des interventions ciblant des maladies spécifiques et les services généraux sont plus probables dans des services et systèmes de santé robustes. Des interventions spécifiques mises en œuvre comme activités parallèles dans des services fragilisés pourraient encore davantage affaiblir leurs capacités de répondre aux besoins de la communauté, d’autant plus lorsque plusieurs IMS interviennent simultanément. Le renforcement des systèmes de santé ne résultera pas de la somme d’interventions mondiales sélectives mais exige une approche globale.

Résumé des auteurs

La prévention des maladies tropicales négligées est récemment passée à échelle en Afrique sub-saharienne, protégeant des populations entières à travers la distribution de masse de médicaments: cinq maladies différentes sont maintenant combattues simultanément avec un paquet de quatre médicaments. Cependant des voix s’élèvent pour dire que, comme d’autres grands programmes de contrôle de maladies spécifiques, la campagne MTN ne parvient pas à renforcer les systèmes de santé et pourrait même affecter négativement la dispensation des soins courants. En 2007, dans une étude de terrain exploratoire au Mali, nous avons observé comment le programme était mis en œuvre dans deux districts ruraux et comment il influençait le système de santé. Nous avons constaté que les effets de la campagne au niveau local variaient selon les services: dans des centres de santé robustes et bien pourvus en ressources humaines, le personnel parvenait à encadrer efficacement la distribution de masse des médicaments tout en assurant les consultations habituelles, et le fonctionnement d’ensemble du service de santé bénéficiait des ressources du programme. Par contre dans des centres de santé plus fragiles, la charge de travail liée au programme perturbait sérieusement l’accès aux soins courants, et nous y avons également constaté des problèmes opérationnels affectant la qualité de la distribution de masse. Des services forts semblent bénéficier tant au contrôle des MTN qu’aux soins généraux.

Introduction

Depuis 2000, les Initiatives Mondiales en matière de Santé (IMS) sont devenues une stratégie d’aide internationale prépondérante, s’appuyant sur des méthodes efficaces pour contrôler des maladies spécifiques, et contribuant de manière substantielle à l’augmentation des ressources pour la santé mondiale [1]. Mais très rapidement des craintes ont été émises: au-delà des bénéfices escomptés pour les maladies visées, ces IMS pourraient en effet éroder les capacités des systèmes de santé à répondre aux besoins généraux de santé [2-7]. Les premières critiques aux IMS portaient surtout sur la distorsion des politiques nationales et sur la création d’organes et processus parallèles constituant un fardeau pour les systèmes de santé [8]. De leur côté, les IMS se rendirent rapidement compte que leurs capacités d’intervention étaient limitées par la faiblesse des systèmes de santé des pays [6].

Alors qu’il est maintenant reconnu qu’IMS et systèmes de santé nationaux s’influencent mutuellement [8,9], partisans respectifs des systèmes de santé et des IMS continuent à véhiculer des points de vues divergents, cadrant en partie dans l’ancien débat horizontal-vertical [10]. Un groupe collaboratif de l’OMS chargé en 2008 de faire le point sur les interactions entre systèmes de santé et IMS a publié ses résultats [9], discutés lors d’une réunion de dialogue politique à Venise en juin 2009 [11]. Le rapport souligne le peu d’évidence disponible pour comprendre les interactions entre IMS et systèmes de santé. Jusqu’à présent, la plupart des études ont traité d’interventions mondiales dans le domaine du contrôle du VIH/SIDA [8]. Les résultats de ces recherches ne sont cependant pas pour autant applicables à d’autres IMS, les différences d’objectifs, de politiques, de structures et de processus opérationnels des IMS étant en effet susceptibles de produire des effets différents sur les systèmes de santé [9]. Une autre limite est que la plupart de ces études concernent le niveau national, alors que l’évidence empirique au niveau de la dispensation des soins reste particulièrement faible [8,9].

Au cours des dernières années, une attention accrue pour les MTN, couplée à l’existence de stratégies de contrôle à relativement faible coût, a suscité d’importante nouvelles initiatives mondiales, dont le Programme Maladies Tropicales Négligées de l’OMS, l'Initiative pour le Contrôle des Schistosomiases (SCI), le Réseau Mondial de lutte contre les Maladies Tropicales Négligées (GNNTD), l’Initiative Maladies Tropicales Négligées (NTDI), et d’autres [12-14]. La stratégie mondiale actuelle de contrôle des MTN met l’accent sur l’administration de masse de médicaments. Se fondant sur des chevauchements géographiques et la co-endemicité, elle vise simultanément jusqu’à cinq MTN (la filariose lymphatique, l’onchocercose, la schistosomiase, les géo-helminthiases et le trachome) avec un paquet de quatre médicaments (ivermectine ou diethylcarbamazine, praziquantel, albendazole ou mebendazole, et azithromycine). Le résultat escompté de la chimiothérapie de masse menée pendant plusieurs années successives est l’élimination ou la réduction des MTN à un taux de prévalence où elles ne constituent plus une menace pour la santé publique. Les arguments en faveur de l’efficience de l’intervention reposent sur l’“intégration” de cinq maladies, mais aussi sur le fait que les médicaments sont donnés par des firmes pharmaceutiques ou disponibles comme génériques, et distribués par des volontaires communautaires [15].

Le Mali, qui avait déjà une expérience de campagnes distinctes pour le trachome et la schistosomiase, fut en 2007 le premier pays à mettre en œuvre ce programme intégré de lutte contre les MTN, avec l’appui financier d’USAID. Les connaissances sur les éventuels effets indésirables de l’administration simultanée de ces médicaments étant jugées insuffisantes, la distribution a été, par précaution, organisée de manière séquentielle sur une période totale de 7 semaines entre avril et juin 2007. Chaque distribution d’un médicament était suivie d’une période de « repos » de deux semaines (semaine 1 : azitromicine, semaine 4: albendazole et ivermectine, semaine 7: praziquantel). En raison de contraintes financières temporaires, la campagne n’a démarré que dans 3 régions, le plan pour le reste du pays étant postposé à fin 2007. La campagne s’est déroulée simultanément dans les 3 régions, concernant 24 districts; dans certains de ces districts, elle a coïncidé avec une campagne de distribution de Vitamine A.

Cette étude analyse les interactions entre le programme de contrôle des MTN et le système de santé du Mali, avec une attention particulière pour le district et le niveau de dispensation des soins. Les districts de santé au Mali sont établis sur un réseau de centres de santé, couvrant chacun une aire de santé définie; le personnel d’un centre de santé est en principe composé d’un infirmier qualifié et de 2 à 4 auxiliaires. Une équipe cadre de district assure le fonctionnement de l’hôpital de référence et apporte un soutien technique aux centres de santé. Le but de cette étude exploratoire était de documenter les processus de mise en œuvre du programme sur le terrain, et d’identifier des effets plausibles, positifs et négatifs, pour le système de santé.

Matériel et Méthode

**Collecte et analyse de données**

Afin d’avoir une meilleure compréhension des interactions entre la campagne MTN et le système local de santé au Mali, nous avons mené une étude qualitative exploratoire, une approche commune lorsqu’il s’agit d’étudier des situations pour lesquelles on ne dispose ni de connaissances préalables étayées ni d’hypothèses précises [16].

Nous avons opté pour l’observation des interactions de la campagne MTN avec les services de santé dans des districts fonctionnant relativement bien, afin d’éviter que nos résultats puissent être attribuables à des défaillances majeures du système local de soins. En ayant recours à un échantillonnage raisonné [16,17], nous avons identifié deux districts ruraux de deux régions différentes, typiques de « bons » districts ruraux en termes d’indicateurs d’output (taux d’utilisation des consultations curatives et taux de couverture des activités préventives).

Notre approche repose sur trois méthodes habituelles de récolte de données qualitatives, à savoir l’observation participante, des interviews en profondeur avec des informateurs clés, et l’analyse de documents [16]. Les interviews sont utiles pour récolter des informations sur des sentiments, des pensées ou des opinions, mais moins pour décrire des évènements, des comportements ou des contextes, les réponses tendant à être déformées par des biais personnels, une conscience insuffisante de la situation, des erreurs de souvenir ou des récits sélectifs [16]. L’observation participante est plus appropriée pour comprendre les contextes, évènements et processus, mais comporte également des limites, dont des comportements atypiques de ceux qui sont observés et la perception sélective de l’observateur [16]. Les documents officiels fournissent des informations utiles, notamment sur les processus tells que planifiés et leurs logiques sous jacente, mais sont sélectifs et ne reflètent pas nécessairement les processus réels de mise en œuvre. Nous avons eu recours à ces différentes méthodes afin de compenser les limites de chacune d’entre elles et de vérifier et trianguler nos résultats [16,17].

L’observation participante a été menée pendant deux semaines en mai – juin 2007 par une chercheuse (AC) compétente en santé publique et familière des systèmes de santé de plusieurs pays d’Afrique sub-saharienne. Dans chacun des deux districts, elle a accompagné le médecin chef de district et / ou le personnel du centre de santé dans leurs activités de suivi de la campagne dans les centres de santé et dans la communauté. Ceci a permis d’observer l’administration de masse de médicaments dans 16 aires de santé (8 aires par district); la sélection de ces aires s’est calquée de manière opportuniste sur l’agenda des districts. L’observation a porté principalement sur des informations de contexte, sur les procédures (la répartition des tâches, le lieu de distribution, le contenu de l’information, le dosage) et sur les comportements du personnel, des distributeurs de médicaments et des membres de la communauté. Lors des observations, la chercheuse avait également recours à des conversations situationnelles, posant des questions ponctuelles et discutant avec les autorités de district et le personnel de santé sur un mode naturaliste et informel [18,19]. En dehors des distributions de médicaments, ont également fait l’objet d’observation participante avec conversations situationnelles une session de « formation de formateurs » pour les équipes de districts au niveau régional, deux réunions d’équipes cadres de district, et une réunion communautaire.

Nous n’avons pas réalisé d’interviews structurés au niveau local en raison des fortes contraintes de temps pour le personnel durant la campagne. Par contre des interviews en profondeur ont été menées avec des informateurs clés, dont dix cadres du Ministère de la Santé et dix représentants de partenaires techniques et financiers connaissant bien le système de santé malien. Ces informateurs clés ont été choisis par échantillonnage par boule de neige [16-17]. Les interviews visaient à récolter des informations sur des processus de la campagne qui ne pouvaient pas être observés directement – tels que les mécanismes de décision au niveau national, la planification et le financement – et à explorer les opinions des informateurs sur les interactions entre le programme de contrôle des MTN et les services locaux de santé.

D’autres informations ont été collectées à partir de la consultation de documents officiels maliens, de recherches sur sites web et une revue de littérature. Nous avons cherché à trianguler nos données tant que possible, vérifiant la même information à des sources différentes.

Les données d’observation et d’interview ont été enregistrées dans des transcriptions de notes de terrain, et analysées séparément par deux chercheuses. L’étude étant exploratoire, les catégories d’analyse ont été essentiellement inductives, mais certaines étaient basées sur la littérature concernant les interactions d’autres IMS avec les systèmes de soins [2-7]. Ces dernières incluaient les processus de mise en œuvre (formation, approvisionnement en médicaments et distribution, monitoring), la distribution des tâches, les effets positifs et négatifs sur la dispensation des soins, et les mécanismes de prise de décision. Lors de l’analyse de ces différents aspects, nous avons porté une attention particulière à l’émergence de tendances récurrentes et aux variations entre situations et/ou informateurs.

Trois sessions de feedback ont eu lieu en novembre 2007 avec les autorités sanitaires des niveaux périphérique, régional et national. Elles ont permis de valider les résultats et de générer des informations complémentaires.

**Déclaration éthique**

La plupart de nos informations sont basées sur des observations de terrain qui n’avaient pas d’effets potentiellement nuisibles pour des patients ou autres personnes vulnérables. La collecte de données a été complétée par des interviews en profondeur avec vingt informateurs clés. Nous leur avons demandé leurs opinions, qui étaient généralement publiques. Nous avons néanmoins demandé un consentement éclairé oral, après avoir expliqué l’objectif et les méthodes de l’étude, et assuré la confidentialité, certains informateurs, cadres du Ministère de la Santé ou d’organisations internationales, préférant ne pas être identifiés. Ce consentement s’est fait en présence d’au moins une personne autre que le chercheur principal. Nous n’avons pas demandé de consentement éclairé écrit, qui eut été inhabituel dans le contexte et aurait pu influencer le processus de l’interview, par ailleurs largement informel. Un autre souci éthique pour cette recherche en systèmes de santé était la gouvernance: les autorités nationales du Ministère de la Santé ont autorisé l’étude, les autorités régionales et de district ont apporté leur support, et les résultats préliminaires ont été partagés et discutés avec les autorités avant toute dissémination plus large. Les problèmes éthiques potentiels ont été examinés avec la Présidente de l’IRB de l’IMT (Anvers) et il a été décidé qu’une revue systématique n’était pas nécessaire. Elle n’était pas exigée non plus par la législation malienne (voir loi n°2009/63/4L), qui régit la recherché biomédicale et non la recherche en systèmes de santé, et n’était pas d’application au moment de l’étude.

Résultats

Nous présentons nos résultats selon le cadre conceptuel des systèmes de santé utilisé par le Groupe de l’OMS sur la maximalisation des synergies positives [9]. Nous examinons tant les effets positifs que négatifs du programme de contrôle des MTN sur la dispensation des soins, les ressources humaines, le système d’information sanitaire, le système d’approvisionnement, le financement et la gouvernance. Ces effets sont résumés dans le Tableau 1.

**La dispensation des soins**

L’accès à la chimiothérapie de masse pour les MTN visées s’est nettement améliorée selon tous les interviewés. Certains informateurs regrettaient cependant que le programme de contrôle se focalise essentiellement sur la distribution de médicaments et secondairement sur la communication pour le changement de comportement, sans inclure d’autres stratégies de contrôle des MTN telles que les soins curatifs (ex : la chirurgie pour le trachome) ou des mesures d’assainissement.

Plusieurs informateurs ont également critiqué la priorité élevée accordée aux maladies ciblées, alors que d’autres problèmes plus communs reçoivent peu d’attention; une de leurs craintes était que la campagne ne mobilise l’énergie et détourne l’attention du personnel des activités de soins habituelles. Ces résultats d’interviews vont dans le même sens que nos observations de terrain: dans la plupart des centres observés, les soins de routine étaient réduits voire totalement interrompus suite à l’absence des infirmiers, non remplacés par d’autres membres du personnel. Il était en effet prévu que, lors de cette première édition du programme de contrôle MTN, les infirmiers chefs des centres de santé consacrent 10 journées de travail entières à des activités de formation et de supervision liées au programme, en plus d’activités de monitoring et d’approvisionnement en médicaments (tableau 2). Le calendrier de la distribution de masse a également obligé les centres de santé à postposer ou réorganiser leurs sessions de vaccination programmées dans la communauté.

Quelques informateurs considéraient que, dans un contexte de faible utilisation de services, la campagne était au moins une façon de rapprocher les services de la population. Nos observations ne suggèrent toutefois pas que la campagne ait eu des effets positifs sur des services non visés. Nous avons observé des occasions manquées pour soins curatifs: des enfants faisant la file pour recevoir des médicaments prophylactiques contre des MTN et présentant de manière évidente d’autres problèmes requérant des soins (ex; abcès ou traumatismes) n’étaient pas identifiés par le personnel présent.

Tous les centres de santé n’ont pas réagi de la même manière à ces interférences: 2 des 16 centres de santé sont parvenus à maintenir un fonctionnement à peu près normal des consultations curatives et des séances de vaccination et ont utilisés la campagne en support du renforcement global de leur centre de santé. Par exemple, un infirmier a transmis le contenu de sa formation MTN aux autres membres de son équipe, un autre a saisi l’opportunité de la formation en MTN des volontaires communautaires pour discuter de problèmes autres que des MTN visées, et la supervision de la campagne au niveau des villages est devenue une occasion d’éducation pour la santé sur d’autres thèmes. Ces deux centres de santé différaient des autres en termes de ressources humaines: tous deux étaient bien pourvus en personnel et n’avaient pas de postes vacants. Ils étaient dirigés par un infirmier qualifié, en poste depuis plus de 5 ans, et réputés pour leur dynamisme, professionnalisme et leadership, tant au niveau régional que central. De plus les taux d’utilisation de ces centres étaient supérieurs à la moyenne nationale (>0.20 nouveaux cas /habitant/an), les taux de couverture préventive étaient considérés bons (>75%), et ils étaient soutenus par une organisation communautaire cohésive.

Nous avons également constaté des différences entre centres de santé dans leurs capacités à mettre en œuvre le programme de contrôle des MTN. En effet nous avons observé des problèmes opérationnels dans tous les centres de santé, excepté les deux plus robustes. Ces problèmes incluaient des erreurs de recensement de population, une mobilisation communautaire faible, des erreurs dans le dosage des médicaments ou encore l’omission du monitoring des effets secondaires. Alors que ces problèmes ne sont pas repérés par le système de monitoring de la campagne MTN dont les indicateurs se limitent au traitement et à la couverture géographique, ils suggèrent néanmoins des problèmes de qualité dans la mise en œuvre de la campagne.

Finalement, plusieurs interviewés ont cité des effets négatifs potentiels de la distribution gratuite de médicaments sur la recherche de soins: puisque les patients malades doivent payer leurs médicaments lors des consultations ordinaires, ils pourraient - c’était du moins leur crainte – attendre l’édition suivante d’une campagne plutôt que de faire la démarche de se faire soigner.

**Les ressources humaines**

Bien que la distribution de médicaments proprement dite soit réalisée par des volontaires communautaires, la campagne MTN a néanmoins augmenté la charge de travail tant pour le personnel des districts que pour celui des centres de santé, chargés de l’approvisionnement en médicaments, du monitoring de la campagne, de la formation et de la supervision.

Plusieurs informateurs considéraient la formation comme un des effets positifs du programme. Une formation en cascade a été organisée, débutant par une “formation de formateurs” au cours de laquelle les coordinateurs des programmes nationaux concernés formaient les autorités de district. La cascade se poursuivait ensuite, les autorités de district formant les infirmiers des centres de santé, qui eux-mêmes formaient les volontaires communautaires. La formation consistait essentiellement à transmettre de l’information sur l’épidémiologie, le diagnostic et le traitement de chacune des maladies visées ; plusieurs participants ont fait remarquer qu’il s’agissait d’une répétition de sessions de formation antérieures.

Pour les activités de formation et de supervision, le personnel recevait des allocations, représentant approximativement une augmentation de 80% du salaire mensuel d’un médecin chef de district (augmentation de 122 000 F CFA pour un salaire moyen de 150 000 F CFA), et une augmentation de 45% du salaire d’un infirmier de centre de santé (augmentation de 46 000 F CFA pour un salaire moyen de 100 000 F CFA). Certains informateurs considéraient ces incitants comme contribuant à la motivation et la rétention du personnel de santé, mais selon d’autres, ces allocations détournaient l’attention du personnel de leurs activités principales, pour lesquelles ils ne reçoivent pas d’allocations.

Les volontaires communautaires recevaient eux aussi des allocations. La plupart de nos informateurs considéraient que c’était une nécessité pour attirer des volontaires. Nous avons été témoins d’une dispute dans un village à propos de la sélection de ces volontaires, ce qui suggère que ce statut était considéré comme enviable. Cependant certains de nos informateurs s’inquiétaient de la pérennité des allocations pour volontaires (supposées être payées par les communautés elles mêmes après le premier tour du programme de contrôle MTN), ainsi que de l’incohérence des montants de allocations entre différents bailleurs, générant des demandes croissantes de la part des volontaires.

**Le système d’information sanitaire**

Les données du recensement réalisé avant la distribution de masse de médicaments peuvent être utilisées à des fins autres que le contrôle des MTN. La campagne a aussi fourni des informations améliorées sur la couverture du traitement prophylactique contre les MTN.

Cependant la campagne a introduit un système parallèle de monitoring et d’évaluation. Au niveau du district, 12 nouveaux formulaires ont été introduits pour la gestion des stocks de médicaments, 15 nouveaux formulaires pour le monitoring et la supervision des activités de la campagne, et un nouveau formulaire pour les complications cliniques de la filariose (Tableau 3). Pour chaque médicament distribué, un rapport était établi par village, un par centre de santé et un par district. Un calendrier spécifique de rapportage permettait de se conformer aux instructions des bailleurs: les données des villages étaient traitées quotidiennement au niveau du centre de santé et transmises au district ; les districts faisaient rapport au niveau régional des résultats de la campagne à un rythme hebdomadaire.

**Le système d’approvisionnement**

La plupart des médicaments de la campagne MTN ont été données par des firmes pharmaceutiques, ce qui a élevé leur disponibilité pour la chimiothérapie préventive à l’échelle du pays. Mais un système parallèle d’approvisionnement en médicaments a été établi pour assurer l’acheminement rapide des médicaments du niveau central vers les niveaux régionaux et de districts. Comme l’espace de stockage au niveau national et régional était insuffisant, des camions ont été loués spécialement pour l’occasion. Des formulaires et procédures de gestion des médicaments, distincts de ce qui existait au niveau national, ont été créés pour la mise en œuvre de la campagne (Tableau 3). Par ailleurs, quelques informateurs ont souligné des déséquilibres en matière de disponibilité en médicaments: alors que l’Azitromicine était distribuée gratuitement durant la campagne, les patients souffrant de trachome n’avaient pas accès à ce médicament en conditions ordinaires, remplacé par une pommade à la tétracycline payante. Un informateur local a signalé des plaintes de la communauté à ce propos.

**Le financement**

Selon les documents officiels [20], le budget prévisionnel pour l’administration de masse de médicaments – coût des médicaments non inclus – était d’approximativement 12 millions US $ répartis sur 5 années, la plupart étant financé par USAID, avec des compléments d’autres agences. Ce budget couvrait la distribution des médicaments (29% du budget), la formation du personnel et des volontaires (26%), la supervision et l’évaluation (17%), l’approvisionnement en médicaments (9%), l’éducation pour la santé (7%), le renforcement institutionnel (10%) et la collaboration intersectorielle (2%). N’étaient pas inclus dans ce budget les frais à charge de l’Etat pour les salaires du personnel et les infrastructures utilisées pour la campagne. Le renforcement institutionnel a consisté principalement en équipement et personnel nécessaires pour l’intervention; plusieurs informateurs ont souligné que le budget ne permettait pas de renforcement institutionnel au-delà des besoins de la campagne. Les investissements en équipement général se sont limités à des motos pour les districts; la proposition de construire un nouveau magasin pour renforcer les capacités limitées de stockage au niveau national n’a pas été approuvée, ni l’acquisition (plutôt que la location) de camions pour l’approvisionnement en médicaments, ce que regrettaient plusieurs informateurs. Tout en reconnaissant la pertinence de l’intervention, ils exprimaient des inquiétudes quant au financement à long terme et la pérennité des résultats de la campagne.

**La gouvernance**

Bien que la décision de mise en œuvre du programme ait été prise par les autorités Maliennes, plusieurs informateurs ont fait remarquer que l’espace de négociation dont disposaient les autorités nationales était restreint, la plupart des décisions étant prises à un niveau supranational par les bailleurs et leurs intermédiaires. En effet le financement USAID n’est pas directement alloué au pays, mais à des sous-bénéficiaires chargés de la mise en œuvre et qui, dans le cas du Mali, étaient l’Initiative Internationale pour le Trachome (ITI), remplacée fin 2007 par Helen Keller International.

Un comité de coordination avec pouvoir de décision a été mis en place parallèlement aux structures existantes au sein du Ministère de la Santé. Il était composé de cadres du Ministère et de personnel ITI. Le plan stratégique de lutte contre les MTN pour 2007-2011 a été élaboré par la Direction Nationale de la Santé. Plusieurs informateurs ont souligné le côté positif de l’élaboration d’un plan unique de contrôle des MTN, qui a stimulé la collaboration entre des coordinateurs de programme auparavant indépendants. Cependant ce plan stratégique devait s’adapter aux exigences des bailleurs et intermédiaires. Dans la mesure où les stratégies étaient largement prédéfinies, les financements assignés et les budgets maintenus serrés dans un but d’efficience, il n’y avait que peu de marges de manœuvre.

Plusieurs informateurs se sont montrés critiques à l’égard des effets de distorsion du programme sur les priorités nationales, tout en reconnaissant l’importance des MTN et la longue expérience du Mali en matière de traitement de masse visant des maladies spécifiques: l’onchocercose depuis 1988, le trachome depuis 2004, la schistosomiase depuis 2005, et des tentatives d’intégrer le traitement de masse pour l’onchocercose, la filariose et les helminthiases démarrées en 2005. Les sites web des bailleurs insistent sur le fait que le programme appuie les efforts des pays et contribue à les faire passer à échelle au niveau national. Mais nos informateurs ont fait remarquer que ces efforts eux-mêmes étaient en partie le résultat d’opportunités de financements externes. Ils considéraient l’accumulation des conditions fixées par les bailleurs comme une entrave à l’allocation des ressources selon les orientations stratégiques nationales.

Enfin, le processus de haut en bas de l’exécution de la campagne MTN a été perçu par de nombreux informateurs comme peu cohérent avec le principe de leadership local du district, central dans la politique de santé Malienne (PRODESS II), et comme interférant avec les activités programmées. En effet, étant donné les brefs délais avec lesquels l’information relative à la campagne était parvenue aux régions et districts, les autorités de districts ont du modifier ou ajourner leur calendrier de supervisions des centres de santé, sans possibilités de négociation.

Discussion

Cette étude est la première à explorer les interactions entre le programme intégré de lutte contre les MTN et le système de santé d’un pays. Elle ouvre des perspectives pour appréhender les effets positifs et négatifs du programme intégré de contrôle des MTN au niveau de la dispensation des soins et des systèmes de districts. Une étude antérieure avait documenté des résistances de la communauté à la distribution gratuite de médicaments contre la schistosomiase et les géo-helminthiases en Ouganda, sans toutefois aborder les effets sur le système de santé [21].

Certaines limites de cette étude doivent être soulignées. La récolte de données a inévitablement été influencée par la présence de la chercheuse et les relations entre elle et les personnes sur le terrain [17]. Il est toutefois plausible que ces effets de recherche aient conduit les autorités et le personnel à vouloir montrer le meilleur de leurs performances et à restreindre leurs commentaires critiques : les biais auraient dès lors plutôt tendance à minimiser plutôt que maximiser les problèmes. Par ailleurs, cette étude qualitative est basée sur un nombre limité d’interviews et d’unités d’observation placées dans leur contexte; les résultats ne sont pas destinés à être généralisés de la même manière qu’en recherche quantitative, bien que le lecteur puisse juger de leur applicabilité dans son propre contexte [17]. L’échantillonnage raisonné [16-17] ne permet pas d’affirmer que les districts et centres de santé observés sont représentatifs pour le pays. Comme nous avons sélectionné des “bons” districts en termes d’indicateurs d’output, nous supposons que les problèmes lies à la campagne ne sont pas plus aigus dans ces districts qu’ailleurs dans le pays, mais ceci nécessiterait vérification. Une autre raison incite à la prudence dans la transposition de nos résultats à d’autres contextes ou pays : la campagne de 2007 au Mali était la toute première à intégrer cinq maladies et peut avoir souffert de problèmes de démarrage, évités dans les éditions ultérieures. Cependant comme la plupart des campagnes MTN financées par USAID sont basées sur des principes et processus similaires à ceux du Mali, notre étude fournit des hypothèses plausibles à tester dans d’autres contextes. L’objectif de notre étude était exploratoire, et d’autres recherches sont nécessaires, tant qualitatives que quantitatives. Nous pensons que nos résultats seront utiles à la formulation de nouvelles questions de recherche.

Une grande partie de notre compréhension actuelle des interactions entre IMS et systèmes de santé repose sur des programmes VIH/SIDA. Bien que le programme de contrôle des MTN apparaisse comme une “petite” IMS par comparaison à d’autres comme le Fonds Mondial ou PEPFAR [1], son analyse ouvre de nouvelles perspectives dans le débat en cours.

Le programme de contrôle des MTN, comme d’autres programmes préventifs, se concentre sur la protection plutôt que sur la réponse aux demandes des patients [22]. Ceci le distingue des Initiatives liées au VIH/SIDA qui visent le passage à échelle pour l’accès de patients individuels aux traitements ARV. Nous avons constaté que le programme intégré contre les MTN n’incluait pas de soins curatifs pour les MTN, mais aussi que la charge de travail supplémentaire et les absences du personnel pour des raisons liées à la campagne perturbaient l’accès aux soins curatifs généraux dans les centres de santé. Une étude de la Fondation Bill and Melinda Gates [23] rapporte un accroissement de la charge de travail pour le personnel des districts en Angola et en Tanzanie résultant des attentes des bailleurs, mais n’évalue pas les effets sur la dispensation des soins. Les connaissances sur les effets qu’a la charge de travail générée par les IMS sur l’accès aux soins dans les centres de santé reste extrêmement limitée [24].

Un résultat capital de notre étude est l’émergence d’une différenciation entre centres de santé d’un même district dans leur capacité à gérer les interférences du programme. Seuls les services les plus résistants, caractérisés par un personnel qualifié, stable et motivé, sont parvenus à maintenir les activités de routine et même à utiliser le programme comme opportunité d’amélioration globale de la qualité. Ce résultat est cohérent avec la notion que les effets positifs des IMS sont plus probables lorsque le système de soins est robuste [9, 25]. Mais il l’étend du système de santé national vers le district et le service de santé, soulignant l’importance des ressources humaines pour la robustesse des services. La recherche future pourrait explorer les relations entre les caractéristiques des ressources humaines et la capacité d’absorption des programmes au niveau des services de santé. Ceci pourrait en effet avoir des implications pour l‘adaptation des programmes aux spécificités des systèmes et service de santé locaux.

D’autres résultats de notre recherche confirment certains effets constatés dans les interactions d’autres IMS avec les systèmes de santé, montrant un mélange d’effets positifs et négatifs. Comme pour d’autres programmes, l’accès aux services visés a été considérablement accru, mais des duplications se sont produites, particulièrement dans l’approvisionnement en médicaments et le monitoring et évaluation [8-9]; ces systèmes parallèles, destinés à augmenter l’efficience de la campagne, ont augmenté la charge de travail et les coûts totaux pour le système de santé. Comme d’autres IMS, le programme de contrôle des MTN a influencé la fixation de priorités [7]: les pressions pour absorber les médicaments offerts et atteindre des résultats rapides de couverture chimio-thérapeutique ont contribué à détourner les énergies d’autres stratégies de contrôle des MTN, dont le traitement et l’hygiène environnementale, et plus généralement du renforcement global du système de santé.

Dans une perspective de systèmes de santé, la question n’est toutefois pas tant de savoir si le bilan des effets d’une IMS particulière est positif ou négatif. Le problème au Mali, comme dans d’autres pays, est l’effet cumulatif d’un grand nombre d’IMS, chacune avec des implications à tous niveaux. A côté des campagnes MTN, les services de santé Maliens sont également impliqués dans les Journées Nationales de Vaccination, les distributions de Vitamine A et de moustiquaires imprégnées, ou encore les campagnes d’’éradication ou d’élimination de la polio, du tétanos ou de la fièvre jaune. Une estimation du temps passé hors de leurs centres de santé par les infirmiers chefs d’un district rural en 2006 a mis en évidence des absences atteignant 54% des jours de travail; la moitié de ce temps était consacré à des campagnes et des formations liées à des programmes verticaux [24]. Comme l’infirmier chef est généralement le seul personnel qualifié pour assurer les soins curatifs de première ligne au Mali, les perturbations dans les horaires de consultation érodent la réactivité du service face aux attentes de la population et la confiance de la communauté dans leur centre de santé [26]. Un autre effet cumulatif est la mobilisation croissante des communautés pour atteindre des objectifs définis d’en haut, aux dépens d’une approche de la participation communautaire visant davantage l’*empowerment*, ou capacitation à prendre en charge leur destinée [21,27].

La nécessité de renforcement des systèmes de santé est de plus en plus largement admise, y compris par les promoteurs du contrôle intégré des MTN [28]. La plupart des IMS affirment contribuer au renforcement des systèmes de santé par l’injection de ressources supplémentaires et le renforcement des capacités, mais ces interventions sont le plus souvent sélectives, visant les fonctions du système essentielles pour la mise en œuvre de leur propre programme [22]. C’est aussi le cas du programme de contrôle des MTN au Mali. La perspective d’ajouter la Vitamine A, des moustiquaires et des vaccins au modèle actuel de la campagne [28] peut contribuer à l’amélioration de la fonction protective des systèmes de santé, mais pas à leur réactivité à la demande de soins curatifs, qui pourrait être encore davantage affaiblie [22].

Le contrôle des MTN dans les communautés vulnérables est une nécessité. Mais le renforcement des systèmes de santé l’est aussi, de manière à répondre adéquatement aux autres problèmes de santé et assurer des résultats pérennes, y compris en matière de contrôle des MTN. Un défi majeur est de trouver des modalités de contrôle de maladies – que ce soit les MTN ou d’autres maladies – sans produire d’effets négatifs sur les systèmes de santé existants. Des connaissances accrues sur les interactions avec le système de santé sont nécessaires pour permettre aux IMS de planifier des effets positifs et minimiser leurs éventuels effets négatifs. Actuellement la priorité est accordée à des interventions à court terme et à effets immédiats, mais des stratégies à long terme sont également nécessaires. Le renforcement des systèmes de santé doit se fonder dans des plans de développement spécifiques aux pays et alignés sur les politiques nationales. Il suppose une approche globale traversant les maladies ou problèmes de santé spécifiques, ainsi que la coordination entre IMS. Par exemple, les formations sur le tas spécifiques à certains programmes devraient être organisées de façon à atténuer les éventuelles interruptions dans la dispensation des soins, mais des investissements sont aussi nécessaires dans la formation initiale du personnel qualifié. L’accumulation d’allocations spéciales liées aux programmes, rendant les interventions ciblées plus populaires que les activités de routine [29], pourrait progressivement être remplacée par une gestion globale des ressources humaines au niveau national et de district. Les systèmes parallèles d’approvisionnement en médicaments devraient être limités aux urgences exceptionnelles, et les investissements redirigés vers le renforcement des systèmes nationaux d’approvisionnement en médicaments.

Tout laisse à penser que les IMS tirent les leçons de l’expérience et modifient progressivement certaines de leurs procédures [8]. Elles montrent aussi une bonne volonté croissante à réduire la fragmentation et remettre en cause les processus [11]. Cependant l’architecture chaotique de l’aide au développement en santé demeure un obstacle majeur pour le renforcement des systèmes de santé. Le progrès vers des systèmes de santé efficaces et inclusifs ne résultera pas le la somme d’interventions sélectives d’IMS.

Références

1. Ravishankar N, Gubbins P, Cooley RJ, Leach-Kemon K, Michaud CM et al.(2009) Financing of global health: tracking development assistance for health from 1990 to 2007. Lancet 373: 2113-2124.

2. Buse K, Walt G (2000) Global public-private partnerships: Part II--What are the health issues for global governance? Bull World Health Organ 78: 699-709

3. Brugha R, Walt G (2001) A global health fund: a leap of faith? BMJ 323: 152-154.

4. Oliveira-Cruz V, Kurowski C, Mills A (2003) Delivery of priority health services: searching for synergies within the vertical versus horizontal debate. Journal of International Development 15: 67-86.

5. Unger, J P, De Paepe P, Green A (2003) A code of best practice for disease control programmes to avoid damaging health care services in developing countries. Int.J Health Plann.Manage 18 Suppl 1: S27-S39.

6. Travis P, Bennett S, Haines A, Pang T, Bhutta Z et al (2004) Overcoming health-systems constraints to achieve the Millennium Development Goals. Lancet 364: 900-906.

7. Shiffman J (2006) Donor funding priorities for communicable disease control in the developing world. Health Policy Plan. 21: 411-420.

8. Biesma RG, Brugha R, Harmer A, Walsh A, Spicer N et al (2009) The effects of global health initiatives on country health systems: a review of the evidence from HIV/AIDS control. Health Policy Plan. 24: 239-252.

9. WHO Maximizing Positive Synergies Collaborative Group (2009) An assessment of interactions between global health initiatives and country health systems. Lancet 373: 2137-2169.

10. Mills A (2005) Mass campaigns versus general health services: what have we learnt in 40 years about vertical versus horizontal approaches? Bull.World Health Organ 83: 315-16.

11. Venice statement: global health initiatives and health systems (2009) Lancet 374: 10-12.

12 Hotez,PJ, Molyneux DH, Fenwick A, Kumaresan J, Sachs SE et al (2007) Control of neglected tropical diseases. N.Engl.J Med. 357:1018-1027.

13. Molyneux DH, Hotez PJ, Fenwick A (2005) “Rapid-impact interventions": how a policy of integrated control for Africa's neglected tropical diseases could benefit the poor. PLoS Med 2 (11): e336.

14. Brady MA, Hooper PJ, Ottesen EA (2006). Projected benefits from integrating NTD programs in sub-Saharan Africa. Trends Parasitol. 22 : 285-291.

15. Fenwick A (2006) Waterborne infectious diseases--could they be consigned to history? Science 313: 1077-1081.

16. Patton MQ (2002) Qualitative research and evaluation methods. Sage Publications, 3^rd^ edition

17. Kuper A, Lingard L, Levinson W (2008) Critically appraising qualitative research. BMJ 2008;337:a1035 doi:10.1136/bmj.a1035

18. Schatzman L, Strauss A.L. (1973) Field research. Strategies for a natural sociology. Prentice Hall Inc., Englewood Cliffs, New Jersey

19. Reeves S., Kuper A., Hodges B.D. (2008) Qualitative research methodologies: ethnography. BMJ 2008;337:a1020 doi:10.1136/bmj.a1020

20. Ministry of Health, National Health Directorate Mali (2007) Plan Stratégique de Lutte contre les Maladies Tropicales négligées 2007-2011. Intégration de la composante chimiothérapie de masse. Available : <http://ntd.rti.org/about/index.cfm?fuseaction=static&label=mali> Accessed 24 June 2010

21. Parker M, Allen T, Hastings J (2008) Resisting control of neglected tropical diseases: dilemmas in the mass treatment of schistosomiasis and soil-transmitted helminths in north-west Uganda. J Biosoc.Sci. 40: 161-181.

22. Marchal B, Cavalli A., Kegels K (2009) Global health actors claim to support health system strengthening: is this reality or rhetoric?" PLoS Med. 6 (4): e1000059.

23. Bill and Melinda Gates Foundation and McKinsey. Global Health Partnerships: assessing country consequences. High-Level Forum on the Health MDGs. Paris 14-15/11/2005. Available: <http://www.hlfhealthmdgs.org/Documents/GatesGHPNov2005.Pdf>

Accessed 11 November 2009

24. Coulibaly Y, Cavalli,A, van Dormael M, Polman K, Kegels,G (2008). Programme activities: a major burden for district health systems? Trop.Med.Int.Health 13: 1430-32.

25. Møgedal S, Stenson B (2000) Disease Eradication: friend or foe to the health system ? Synthesis Report from field studies on the Polio Eradication Initiative in Tanzania, Nepal and the Lao People's Democratic Republic. WHO Geneva, Department of Vacccines and Biologicals

Available: <http://209.85.229.132/search?q=cache:BK0t9mMeKxMJ:www.who.int/vaccines-documents/DocsPDF00/www552.pdf+Mogedal+WHO+vaccines+and+biologicals&cd=9&hl=fr&ct=clnk&gl=be>

Accessed 11 November 2009

26. Segall M (2003) District health systems in a neoliberal world: a review of five key policy areas. Int.J Health Plann.Manage 18: S5-S26.

27. Perez D, Lefevre P, Romero MI, Sanchez L, De Vos P et al. (2009) Augmenting frameworks for appraising the practices of community-based health interventions. Health Policy Plan 24: 335-341.

28. Hotez PJ, Fenwick A, Savioli L, Molyneux DH (2009) Rescuing the bottom billion through control of neglected tropical diseases. Lancet 373: 1570-1575.

29. Hanefeld J, Musheke M (2009) What impact do Global Health Initiatives have on human resources for antiretroviral treatment roll-out? A qualitative policy analysis of implementation processes in Zambia. Hum Resour.Health 7:8 doi:10.1186/1478-4491-7-8

**Tableau 1. Effets des interactions entre contrôle MTN et système de santé**

| **Point d’interaction** | **Effets positifs** | **Effets négatifs** |
| --- | --- | --- |
| Dispensation des soins | - Accès accru à la chimiothérapie préventive - Dans les centres de santé robustes: campagne comme opportunité de renforcer les capacités et la réactivité du centre | - Occasions manquées de soins curatifs - Dans les centres de santé fragiles: absences du personnel qualifié du centre = interruption des activités générales; problèmes opérationnels dans la mise en œuvre de la campagne - La perspective de distribution gratuite de médicaments pourrait affecter l’utilisation des services de santé |
| Personnel | - Recyclage sur les MTN - Allocations pour médecins chef de district (81% du salaire mensuel), infirmiers de centre de santé (46% du salaire mensuel) et volontaires communautaires - Allocations susceptibles de contribuer à la rétention du personnel | - Charge de travail accrue pour le personnel du district et des centres de santé - Bénéfices de la formation possiblement limités lorsqu’il d’agit de transmission d’informations connues - Allocations pouvant détourner l’attention du personnel de ses activités principales - Incertitudes quant à la pérennité d’allocations aux volontaires communautaires |
| Système d’information sanitaire | - Données disponibles sur la couverture du traitement MTN - Données de recensement utilisables à des fins autres que les MTN | - Système parallèle de monitoring et évaluation (au total 25 nouveaux formulaires au niveau du district) ; calendrier spécifique pour l’envoi des rapports |
| Système d’approvisionnement | - Augmentation de la disponibilité de médicaments MTN au niveau national | - Système parallèle d’approvisionnement: location de transport spécial (camions) - Déséquilibre: certains médicaments distribués en campagne ne sont pas disponible pour les soins curatifs de routine |
| Financement et Gouvernance | - Augmentation des fonds disponibles - Renforcement des efforts des pays dans le contrôle des MTN - Elaboration d’un plan unique de contrôle MTN; stimulation de la coordination entre programmes | - Marges de manœuvre restreintes: budgets et financements assignés - Renforcement institutionnel limité et sélectif - Incertitudes sur la pérennité à long terme - Accumulation de conditions des bailleurs détournant les orientations stratégiques nationales - Mécanismes de décision de haut en bas interférant avec les plans et calendriers régionaux et de district |

**Tableau 2: Absences des infirmiers chefs en raison d’activités liées à la campagne MTN**

| **Activité** | **N° jours de travail** |
| --- | --- |
| Participation à la formation de formateurs | 4 |
| Formation distributeurs communautaires | 3 |
| Supervision de la distribution | 3 |
| Définition besoins médicaments / gestion | Non mesurable |
| Monitoring effets indésirables médicaments | Non mesurable |
| Collecte et transmission des données | Non mesurable |
| Mobilisation communautaire | Non mesurable |
| Phase de restitution | Non mesurable |
| TOTAL | >10 |

**Table 3: Nouveaux formulaires pour activités liées à la campagne MTN**

| **Type de formulaire** | **Description** | **Nombre** |
| --- | --- | --- |
| Gestion des stocks de médicaments | Fiches d’inventaire | 3 |
|  | Modification des stocks | 3 |
|  | Réception | 3 |
|  | Dotation | 3 |
| Monitoring de la distribution de médicaments | Rapports district | 3 |
|  | Rapports aires | 3 |
|  | Rapports villages | 3 |
| Supervision | Communauté | 3 |
|  | District | 3 |
| Rapport de complications cliniques pour la filariose lymphatique |  | 1 |
| **TOTAL** | **25** | |
